# Beyond segmentation accuracy: understanding model reliability and failure modes in ultrasound image analysis

**DOI:** 10.3389/fmedt.2026.1918432

**Published:** 2026-09-23

**Authors:** Agathe Bricout, David Hardman, Scott Inglis, Stephen Pye, Carmel M. Moran

**Affiliations:** 1 Institute for Neurological and Cardiovascular Research, University of Edinburgh, Edinburgh, United Kingdom; 2 Centre for Medical Informatics, University of Edinburgh, Edinburgh, United Kingdom; 3 Medical Physics, NHS Lothian, Edinburgh, United Kingdom

**Keywords:** explainable AI, interpretability, medical imaging, segmentation, ultrasound

## Abstract

Segmentation of ultrasound images is complex, and although deep learning has improved automation, trust remains limited, restricting adoption. Interpretability methods may help reveal model behaviours and failure modes, supporting more trustworthy AI systems. A U-Net with a ResNet-18 backbone was trained to segment pipes in images of the Edinburgh Pipe Phantom acquired using ten ultrasound probes (*N* = 162). Performance was assessed using five-fold cross-validation. Predictive entropy and variance across 20 Monte Carlo dropout forward passes were used as approximations of data-related and model-related uncertainty. Calibration was assessed using the Brier score and expected calibration error (ECE). Input-gradient saliency and Grad-CAM maps from four encoder stages and the final decoder block were evaluated. For exploratory prospective quality control, complete empty-mask failures were assessed directly from model output, while non-empty predictions were classified retrospectively using reference-based performance criteria and reference-free uncertainty and morphological features were evaluated as candidate indicators. The model achieved a Dice score of 0.81 ± 0.03 and an HD95 of 11.98 ± 3.39. Seven segmentation errors were identified: three complete failures in which no foreground mask was predicted and four non-empty predictions in which the incorrect pipe was segmented. Dice score was associated with the Brier score (Spearman *ρ* = −0.96) and ECE (*ρ* = −0.72), whereas predictive entropy and Monte Carlo dropout variance showed weak associations with segmentation performance (|*ρ*| < 0.4). In selected failure examples, uncertainty and activation sometimes overlapped the reference pipe region despite incorrect segmentation. Complete empty-mask failures were deterministically identifiable from the model output, whereas the evaluated reference-free indicators showed limited ability to identify poor-quality non-empty segmentations. Within this phantom dataset and model configuration, calibration metrics were more closely associated with segmentation quality than the evaluated uncertainty approximations. Attention and uncertainty maps provided complementary descriptive information about selected errors, but indicated spatial sensitivity rather than causal model reasoning. Simple output checks enabled deterministic identification of complete segmentation failures; however, the evaluated reference-free indicators were insufficient for validated prospective identification of subtler non-empty errors. These findings support multimodal model evaluation for EPP segmentation while emphasising the need for validation on independent datasets, devices and architectures in future studies.

## 1 Introduction

Ultrasound is the most widely used medical imaging modality in the world beyond traditional radiography applications (1, 2), due its real-time imaging capacities, relatively low cost and non-ionising nature. It relies on the emission and reception of high-frequency sound waves, creating an image based on the acoustic interactions within the tissue (3).

Quality assurance and performance assessments are performed at different timepoints of an ultrasound scanner's lifespan, ensuring that it is fit for purpose. This process often relies on medical physicists scanning test-objects—also known as phantoms, which offer predictable and controlled test environments. The images acquired are then analysed and compared to established guidelines (4), a time-consuming process which could be accelerated through automated segmentation and subsequent analysis.

Image segmentation is a routine medical image analysis task also performed by sonographers and radiologists alike, enabling information collection about different internal organs and structures, to allow for automated area or volume calculations as well as computer-aided diagnosis (5). Ultrasound segmentation is, however, inherently challenging (6).

The different acoustic interactions of the ultrasound beam within tissue create characteristic speckle, shadows obscuring organs, possible artifacts and result in a low signal-to-noise ratio. Multiple deep-learning (DL) approaches have been developed to facilitate automated segmentation of ultrasound images (7), primarily using convolutional neural networks (CNNs) such as U-Net (8) or ResNet (9).

Due to the recent progress in DL research (10–12) and the push towards computer-aided diagnostics to alleviate pressure on healthcare professionals (13), there is growing concern surrounding the trustworthiness of the ML predictions and clinical implications of errors (14, 15). This is exacerbated by the “black-box” problem—the inability to understand how the model reached its prediction as only the output is known for a given input (16).

In this study, segmentation performance refers to agreement between model-predicted segmentation masks and the corresponding reference masks; calibration refers to agreement between predicted probabilities and observed pixel labels; uncertainty refers to estimated ambiguity or variability in a prediction; and reliability refers to consistent behaviour under the conditions evaluated. Trustworthiness is used as a broader concept that may incorporate performance, calibration, robustness, transparency and appropriate use. These terms are therefore not used interchangeably.

Interpretability aims to improve clinical trust in AI for medical imaging by using different approaches (17, 18) to shed light on the internal processes of the model. It can also assess model confidence in its predictions through epistemic and aleatoric uncertainty calculations, as well as calibration measurements. Predictive entropy and Monte Carlo dropout can be used as approximate uncertainty indicators, whereas the Brier score and expected calibration error assess probabilistic calibration. Grad-CAM (19) and other saliency maps (20) are used to highlight which pixels contributed most to the model's predictions and whilst such maps should not be interpreted as proof of causal reasoning, they can support systematic investigation of model behaviour and failure modes (21).

The resolution integral, first described in 2004 (22), is a dimensionless single figure-of-merit representing the overall imaging performance of greyscale ultrasound scanners. It enables direct comparison between scanners and between different stages of a scanner's lifecycle. Resolution integral measurements can be obtained using the Edinburgh Pipe Phantom (EPP), a purpose-built test object containing wall-less, fluid-filled pipes suspended in tissue-mimicking material (22, 23). The conventional method requires an operator to identify the most superficial and deepest visible extent of each pipe and is therefore time-consuming and potentially observer-dependent. A deep-learning method was previously developed to automate these measurements through pipe segmentation in Bricout et al. (24), but its calibration, uncertainty, attention patterns and failure modes were not examined.

The present study does not propose a new explainability algorithm. Instead, its contribution is an integrated evaluation of a previously developed EPP segmentation model using complementary performance, calibration, uncertainty and attention analyses. The objectives were to determine whether these measures provide information beyond conventional segmentation metrics, to examine complete and partial failures, and to assess whether simple reference-free indicators could support image-level quality control. All conclusions are restricted to the EPP dataset and model configuration evaluated.

## 2 Methods

### 2.1 Dataset

An EPP was scanned with a GE Logiq E10, a Mindray A20 and a Samsung R20 ultrasound scanner using a variety of curvilinear, linear and phased array probes (*N* = 10). A trained operator acquired a total of 162 images by following the conventional resolution integral measurement protocol (see Figure 1) (25). For each available scanner-probe combination, superficial and deep visibility measurements were performed for each visible pipe. These measurements were acquired as separate ultrasound images rather than as different frames from the same sweep. Each acquisition was independently optimised for the measurement being performed, including adjustment of the imaging field and scanner settings required to visualise either the superficial or deep extent of the pipe. Consequently, no duplicate images or repeated frames were included for a given scanner-probe-pipe-measurement-position combination.

**Figure 1:**
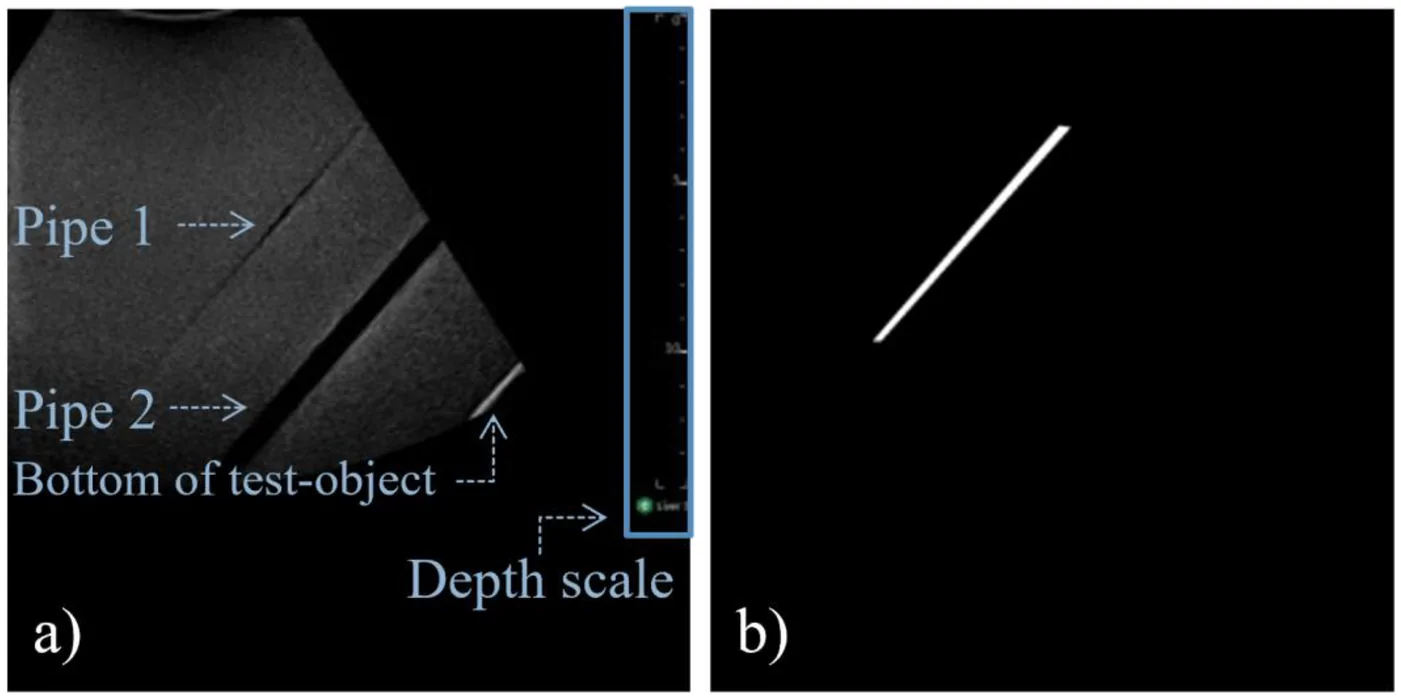
Example images of a) a scan acquired on the EPP with b) the corresponding expert-labelled mask. Note that only the top pipe (Pipe 1) is of interest in this image despite two being visible.

To represent the two possible orientations in which an operator may hold the probe, half of the images were randomly selected and flipped horizontally. This was treated as fixed preprocessing rather than online augmentation: the flipped image replaced, rather than supplemented, its original counterpart. Each image was then cropped to a square region containing the target pipe and depth scale and resized to 256 × 256 pixels.

A ground truth binary mask was created for each image by manually segmenting the pipes of interest using the VIA annotation software (26). The pipe of interest was identified using the corresponding visual resolution integral measurement, and the segmented pipe extent was checked against the visually measured pipe length. The resolution integral values obtained from the manually segmented images differed from the corresponding visual measurements by 0.9 ± 0.3% (Figure 1b), which was within the previously reported 3% repeatability of resolution integral measurements (27). Masks were created before model training and without access to model predictions. A separate boundary-level inter-rater annotation study was not performed.

Stratified five-fold cross-validation was used during training and testing to assess model performance and ability to generalise to unseen data (28). The 162 images were divided into five mutually exclusive outer folds containing 33, 33, 32, 32 and 32 images, respectively (see Figure 2a). Stratification was performed according to the individual ultrasound probe used for acquisition, such that the relative contribution of each of the ten probes was approximately balanced across the five folds (Figure 2). Consequently, the test folds contained unseen images but not necessarily unseen probes or scanners. Superficial and deep measurements from the same scanner-probe-pipe combination were treated as separate image-level observations during the original stratification and were not explicitly constrained to the same fold. In each iteration, four outer folds were used for model development, and the remaining fold was reserved exclusively for testing. The test fold was not used for learning-rate adjustment, checkpoint selection or quality-control threshold exploration (see Figure 2b).

**Figure 2:**
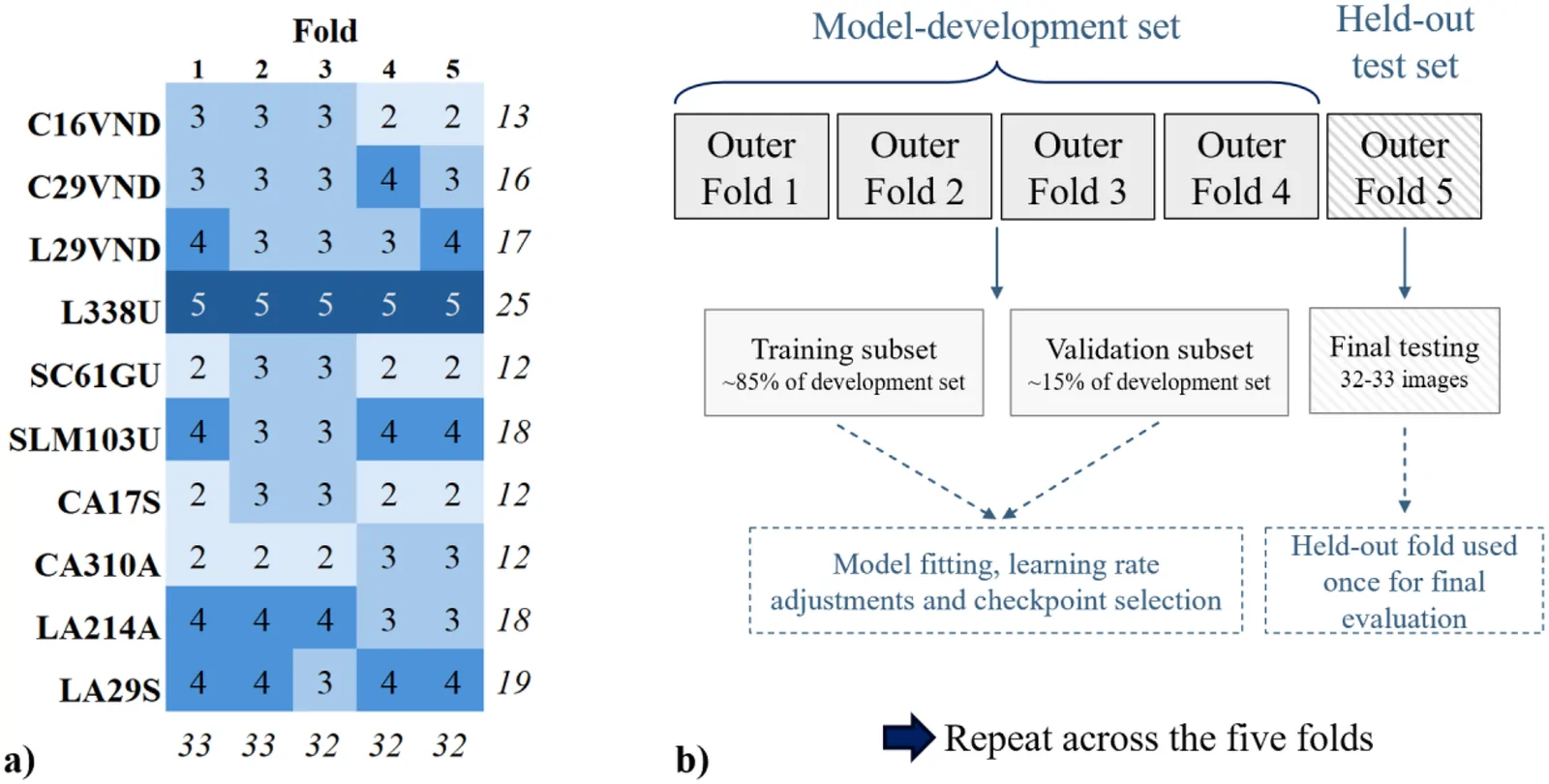
Distribution of images and outer cross-validation design. **(a)** Number of images acquired using each of the ten individual ultrasound probes assigned to each held-out outer fold. Cell annotations indicate image counts, with row and column totals showing the number of images per probe and per test fold, respectively. **(b)** Example of one cross-validation iteration, in which four outer folds formed the model-development set and the remaining fold was held out for final testing. The held-out fold rotated across the five iterations so that every image contributed once to testing.

Within the four-fold development set, a second stratified split was performed to create training and validation subsets. Approximately 15% of the development images were assigned to validation, while preserving the probe distribution as closely as possible. The validation subset was used to monitor convergence, adjust the learning rate and select the model checkpoint with the highest validation Dice score. Predictions from the five held-out test folds were then combined so that each image contributed once to the final evaluation. This strategy evaluated generalisation to unseen images acquired using probes represented during model development, rather than to completely unseen probes.

### 2.2 Model architecture

A modified U-Net architecture with a ResNet-18 backbone was used as the segmentation model assessed throughout this study. This pairing was selected because it achieved the best performance for EPP segmentation in the authors' previous comparison of conventional U-Net and ResNet models with U-Net models using ResNet encoders of different depths (24). A quantitative summary of that comparison is provided in Supplementary Material A. The present study evaluated the reliability and interpretability of this selected model rather than repeating architecture optimisation.

The model was implemented with the *segmentation_models_pytorch* (29) package. To facilitate Monte Carlo dropout, a two-dimensional dropout layer (*p* = 0.3) was applied independently to each encoder feature map prior to decoding. The network accepted single-channel greyscale ultrasound images and generated two-class logits for pipe and background. Training used cross-entropy loss with label smoothing (*ε* = 0.1) (30), the *Adam* optimiser with an initial learning rate of 0.001 (31), and a ResNet-18 encoder initialised with weights pretrained on ImageNet (31).

Monte Carlo dropout was selected as a computationally accessible approximation of epistemic uncertainty because it generates a distribution of predictions from repeated stochastic forward passes through a single trained network. Retaining dropout during inference can be interpreted as approximate Bayesian inference, with greater between-pass variability indicating greater sensitivity to the sampled model configuration (32). This approach required less training and storage than independently trained deep ensembles. Pixel-wise Monte Carlo dropout variance was calculated across 20 stochastic forward passes; it is therefore described as an approximation rather than a direct measurement of epistemic uncertainty (see Figure 3).

**Figure 3:**
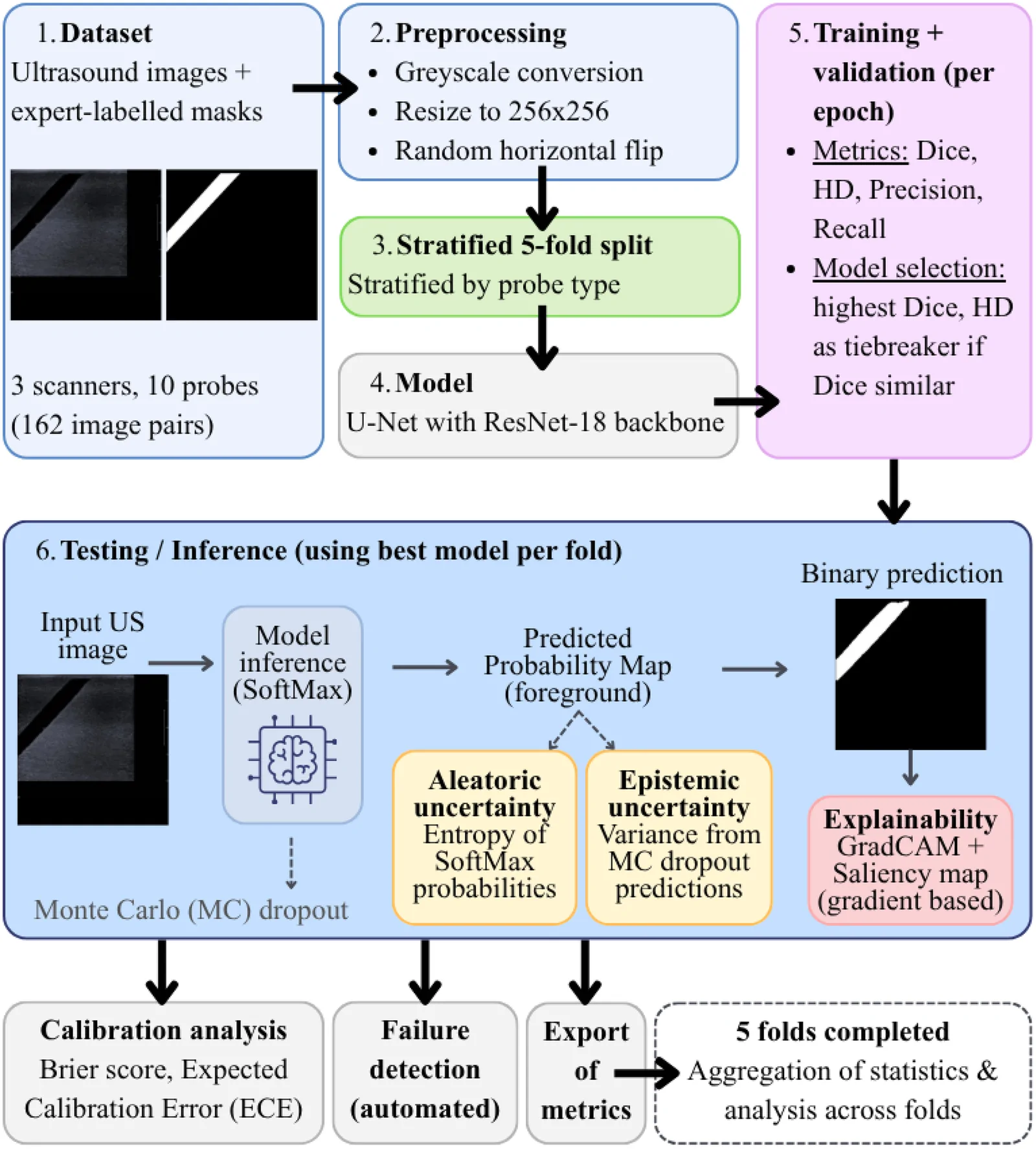
Study pipeline, including dataset preprocessing, model development, checkpoint selection, held-out inference and post-processing.

Models were trained for 50 epochs. After each epoch, validation Dice score (DS) (33) and the 95th-percentile Hausdorff distance (HD95) (34) were calculated. A checkpoint was saved when validation DS reached a new maximum; HD95 was used as a tiebreaker when the difference in DS was less than 0.01. A *ReduceLROnPlateau* (35) scheduler reduced the learning rate by a factor of 0.5 after three epochs without improvement in validation DS.

Single-forward-pass inference time was measured on 256 × 256-pixel images using a batch size of 2. Training and primary evaluation were performed on a workstation with an NVIDIA GeForce RTX 4,090 GPU (24 GB VRAM), an AMD Ryzen Threadripper PRO 5965WX 24-core processor and 251.5 GB RAM. CPU-only inference was also evaluated on a laptop with an AMD Ryzen AI 9 HX 370 processor and 31.1 GB RAM. The timing benchmark did not include the 20 stochastic passes required for Monte Carlo dropout, saliency-map generation, Grad-CAM generation or post-processing.

### 2.3 Interpretability

Two uncertainty-related quantities were calculated from the model outputs. Predictive entropy was derived from the SoftMax probabilities and used as an approximation of data-related ambiguity; it was not treated as a pure or direct measurement of aleatoric uncertainty. Monte Carlo dropout variance was calculated across the 20 stochastic predictions and used as an approximation of model-related, or epistemic, uncertainty (36). Both were visualised as pixel-wise maps, with larger values indicating greater entropy or between-pass variability.

Input-gradient saliency and Gradient-weighted Class Activation Mapping (Grad-CAM) were used to examine spatial sensitivity at different resolutions. For each input, the gradient of the summed foreground-class score with respect to the image was calculated by backpropagation. The absolute gradients (Equation 1) were normalised to ([0,1]) to produce a pixel-level saliency map. These maps indicate sensitivity of the output to local intensity changes and were not interpreted as causal explanations.

$$\begin{array}{c} S(x,y)=\left|\frac{\partial Y}{\partial I(x,y)}\right| \end{array}$$ (1)

where I(x,y) is the input pixel intensity, *Y* is the summed foreground-class score, and S(x,y) is the saliency value.

Grad-CAM was applied to the final residual block of each of the four encoder stages and to the last decoder block. For each image, explanations were generated for the pipe class using the predicted segmentation mask as the target region. Gradients of the target output with respect to the selected feature maps were spatially averaged to obtain channel importance weights (see Equation 2a), which were then combined with the feature maps to produce a localisation heatmap (Equation 2b). Maps were normalised and visualised using a colour scale for qualitative assessments of model attention.

$$\begin{array}{c} \alpha_{k}=\frac{1}{Z}\underset{i}{\sum }\underset{j}{\sum }\frac{\partial Y}{\partial A_{ij}^{k}} \end{array}$$ (2a)

$$\begin{array}{c} L_{GradCAM}=ReLU\left(\underset{k}{\sum }\alpha_{k}A^{k}\right) \end{array}$$ (2b)

where $A^{k}$ is the feature map *k*, *Y* is the target output, $\alpha_{k}$ is the importance weight, and $L_{GradCAM}$ is the Grad-CAM heatmap.

The maps were inspected qualitatively to determine whether elevated activation or uncertainty overlapped physically meaningful structures, such as the target pipe and its boundaries, or unrelated speckle and artefacts. The selected examples were treated as illustrative rather than representative or causal evidence.

### 2.4 Quantitative analysis

The model's catastrophic failure rate was defined as the number of held-out images for which no foreground pipe region was predicted despite a non-empty reference mask. For non-failed inferences, segmentation agreement was assessed using DS, HD95, precision, recall, Jaccard coefficient (JC), relative absolute volume difference (RAVD) and average surface distance (ASD). Empty image-mask pairs and failed inferences were handled separately as surface-distance metrics are undefined when either the reference or predicted mask is empty. For images containing a reference foreground but no predicted pipe, DS, JC and recall were assigned a value of 0, while HD95 and ASD were recorded as undefined and excluded from summaries of boundary-distance metrics. Surface-distance metrics were calculated normally for non-empty predictions with no overlap with the reference mask, including wrong-pipe segmentation errors; they were considered undefined only when either mask was empty. Any boundary-distance metrics were calculated in pixels.

An exploratory image-level quality-control analysis was then performed to determine whether segmentation errors and other poor-quality outputs could be flagged automatically for review. Whilst empty predictions corresponding to complete segmentation failures could be identified at inference with a deterministic flag, non-empty predictions required a more advanced quality-control approach. Morphology-based (bounding-box fill fraction, predicted area deviation, component count deviation) and uncertainty-based (mean entropy) image-level indicators were evaluated, with the Dice score and HD95 treated as retrospective indicators of segmentation quality. Reference-free quality-control indicators were evaluated only among non-empty predictions, where poor segmentation quality was retrospectively defined as Dice <0.50 and/or HD95 > 20 pixels.

For the morphology indicators, predicted foreground area was expressed relative to the median predicted foreground area in the corresponding development set:

$$\begin{array}{c} \mathrm{PredAreaRati}o_{i}=\frac{A_{i}}{\tilde{A}}\; \end{array}$$ (3a)

where $A_{i}$ is the predicted foreground area for image *i* and $\tilde{A}$ is the median predicted foreground area. Morphological deviation was defined as the absolute natural logarithm of the predicted area ratio, with higher values therefore indicating an unusually small or large predicted segmentation. The bounding-box fill fraction was defined as the ratio of predicted pixels to the area of the smallest rectangular box enclosing them:

$$\begin{array}{c} B\mathrm{BoxFillFractio}n_{i}=\frac{A_{i}}{A\mathrm{bo}x_{i}} \end{array}$$ (3b)

where $A_{i}$ is the predicted foreground area for image *i* and $A\mathrm{bo}x_{i}$ is the area of the corresponding bounding rectangular box of height $h=y_{\max}-y_{\min}+1$ and width $w=x_{\max}-x_{\min}+1$, with (x,y) denoting pixel coordinates.

Threshold sensitivity analyses were performed separately for each reference-free indicator. For each held-out fold, candidate thresholds were explored using predictions from the remaining four folds. Thresholds achieving 100% sensitivity were prioritised, with the threshold providing the highest specificity selected; where multiple thresholds produced the same specificity, the threshold yielding the lowest review rate was retained. If no threshold achieved 100% sensitivity, the threshold maximising balanced accuracy was selected. The selected threshold was then applied without modification to the remaining fold. This procedure was repeated so that each fold served once as the held-out quality-control evaluation set. Predictions from the five held-out evaluations were pooled to calculate overall sensitivity and specificity. Sensitivity and specificity were calculated from the pooled confusion matrix and are reported with 95% Wilson confidence intervals. The thresholds selected across the five iterations are reported as mean ± standard deviation. Sensitivity curves and analyses for additional metrics are provided in Supplementary Material B.

The Brier score (37) and Expected Calibration Error (ECE) (38) were chosen to assess the correspondence between predicted probabilities and reference labels. Both were calculated on a pixel-by-pixel basis for each image. The Brier score is the mean squared difference between the predicted foreground probability and the observed binary label (Equation 4).

$$\begin{array}{c} \mathrm{Brier}=\;\frac{1}{N}\overset{N}{\underset{i=1}{\sum }}{\left(\;p_{i}-y_{i}\right)}^{2}\; \end{array}$$ (4)

where $p_{i}$ is the normalised predicted probability, $y_{i}$ is the actual outcome (0 background; 1 pipe) and *N* is the number of predictions evaluated (i.e., number of pixels).

The ECE describes the difference between average predicted probability and observed frequency. For each image, pixel predictions were assigned to ten probability bins. Within each bin, the mean predicted probability was compared with the observed foreground frequency, and the absolute differences were averaged using the number of pixels in each bin as weights (Equation 5).

$$\begin{array}{c} ECE=\;\overset{M}{\underset{m=1}{\sum }}\frac{\left|B_{m}\right|}{N}|\mathrm{acc}(B_{m})-\mathrm{conf}(B_{m})| \end{array}$$ (5)

where *M* = 10 is the number of bins, $B_{m}$ is the number of samples in a specific bin, *N* is the total number of samples (i.e., number of pixels), $\mathrm{acc}(B_{m})$ is the accuracy rate of the sample in that bin and $\mathrm{conf}(B_{m})$ is the average predicted probability (confidence) of the samples in that bin.

Lower Brier scores and ECE values indicate closer agreement between predicted probabilities and observed labels, although neither metric has a universal threshold defining acceptable calibration for this application.

To supplement the qualitative visualisations, two image-level attention measures were calculated from each normalised Grad-CAM and saliency map. Attention localisation was defined as the proportion of total activation within the reference pipe mask. Attention entropy described the spatial dispersion of the normalised activation distribution, with lower values indicating more concentrated activation. Spearman correlations between each attention measure and DS were calculated separately for each targeted layer and held-out fold. These analyses were descriptive and were not interpreted as evidence that the highlighted regions causally determined the prediction.

## 3 Results

### 3.1 Training assessment

Training loss decreased consistently across all five folds, while validation loss generally decreased before reaching a plateau (see Figure 4). Greater variability and limited late-stage divergence were observed in some validation curves, suggesting mild fold-specific overfitting. Checkpoint model selection based on validation performance prevented later epochs with poorer generalisation from being used for held-out testing, however the curves do not exclude overfitting or establish generalisation beyond the sampled probe and scanner distribution.

**Figure 4:**
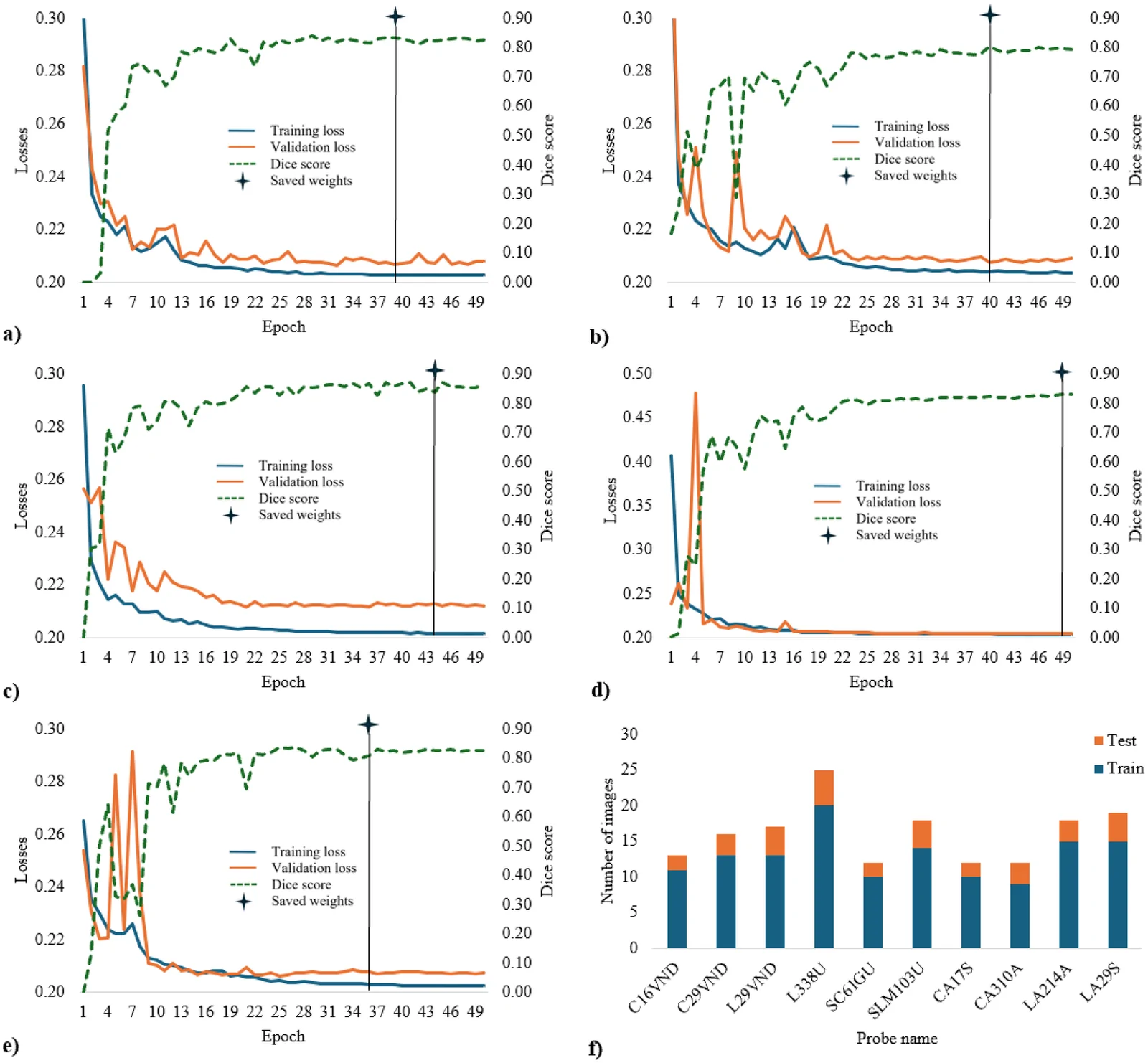
Training loss, validation loss and validation dice score across epochs for folds 1–5 **(a–e)**. The black star marks the selected checkpoint. Panel **(f)** shows the proportion of images from each probe used in the model-development and held-out test sets.

### 3.2 Segmentation performance

Mean single-forward-pass inference time was 0.003 ± 8.289e-5 s per image on the high GPU workstation and 0.036 ± 0.001 s on the CPU-only laptop (162 images, batch size = 2). These values exclude the additional forward passes and map-generation steps required for the complete interpretability pipeline. Held-out segmentation performance was similar across folds, with an overall DS of 0.81 ± 0.03 (Table 1). Three complete failures occurred among 162 images (1.85%).

**Table 1:** Held-out segmentation performance by outer fold.

Fold	Failure	DS	HD95	Precision	Recall	JC	RAVD	ASD
1	1 (/33)	0.85 [0.04]	8.77 [6.64]	0.89 [0.04]	0.83 [0.06]	0.75 [0.05]	−0.05 [0.09]	2.18 [2.22]
2	1 (/33)	0.79 [0.09]	8.74 [6.01]	0.86 [0.09]	0.77 [0.10]	0.71 [0.09]	0.05 [0.37]	4.60 [4.78]
3	0 (/32)	0.79 [0.06]	14.77 [8.06]	0.84 [0.07]	0.76 [0.08]	0.67 [0.07]	−0.10 [0.09]	5.21 [5.01]
4	1 (/32)	0.78 [0.08]	16.40 [10.18]	0.82 [0.06]	0.80 [0.09]	0.68 [0.08]	0.03 [0.14]	4.54 [3.84]
5	0 (/32)	0.82 [0.06]	11.36 [7.85]	0.82 [0.07]	0.85 [0.07]	0.72 [0.07]	0.06 [0.14]	3.70 [2.85]
*Overall*	*3* (*/162)*	*0.81 [0.03]*	*11.98 [3.39]*	*0.85 [0.03]*	*0.80 [0.03]*	*0.71 [0.03]*	*0.00 [0.08]*	*4.04 [1.67]*

Values are means with 95% confidence-interval half-widths in square brackets and are calculated among successful inferences following normal-theory unless otherwise stated. HD95 and ASD are reported in pixels. DS, Dice score; HD95, 95th-percentile Hausdorff distance; JC, Jaccard coefficient; RAVD, relative absolute volume difference; ASD, average surface distance.

### 3.3 Model attention and uncertainty visualisation

Figure 5 presents three retrospectively selected examples spanning different outcomes: a good segmentation (DS = 0.89; HD95 = 1.41), an intermediate segmentation (DS = 0.86; HD95 = 5.21) and an erroneous segmentation (DS = 0.00; HD95 = 73.11). Predicted and reference masks are overlaid on the ultrasound images, with overlap shown in yellow, reference-only regions in green and prediction-only regions in red. Predictive-entropy and Monte Carlo dropout-variance maps are also shown. Layer-wise Grad-CAM and input-gradient saliency maps for the good and erroneous cases are presented in Figures 6,7, respectively.

**Figure 5:**
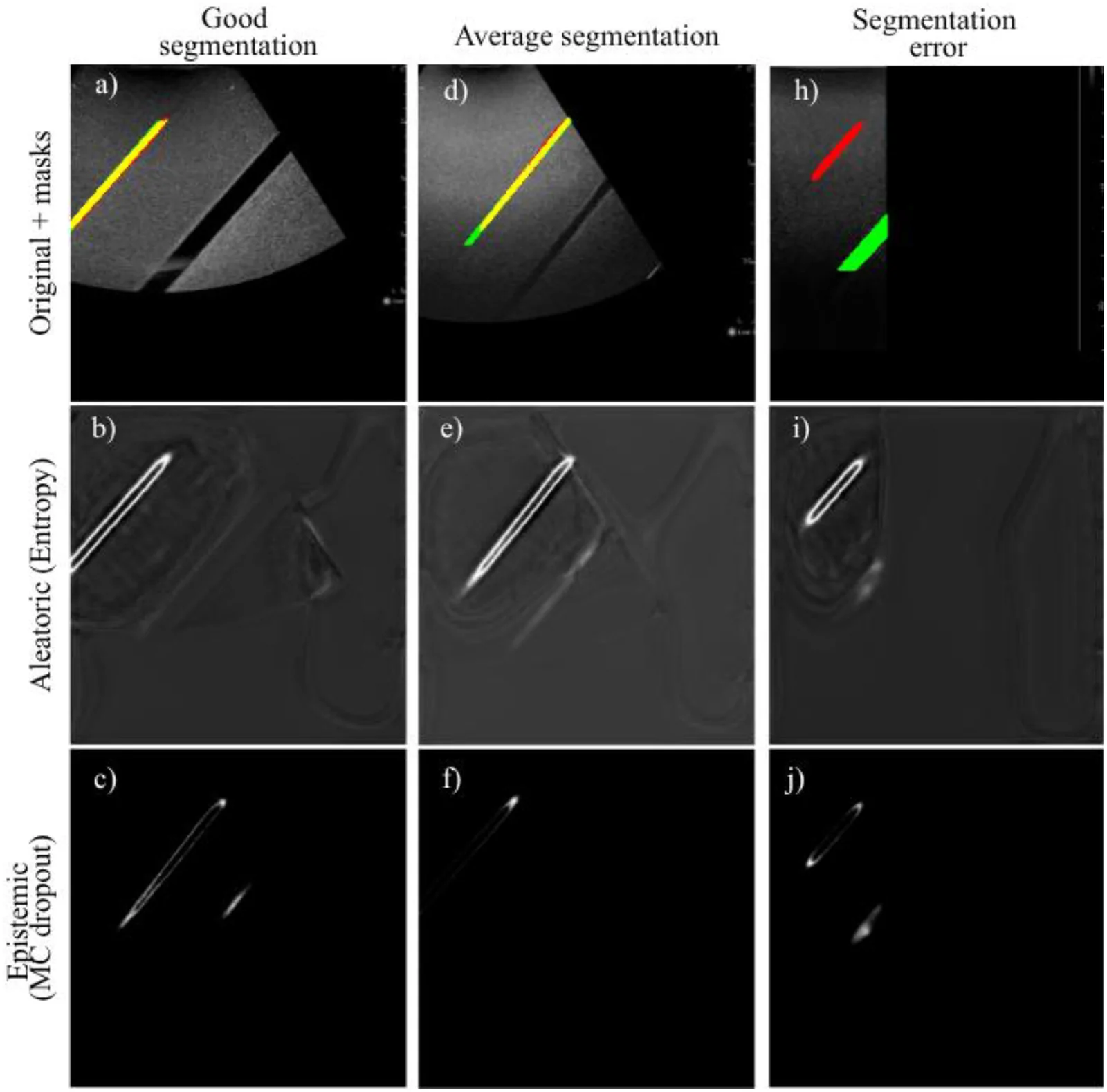
Predicted masks, reference masks and uncertainty maps for three illustrative segmentation outcomes (**a-c** “Good segmentation”, **d-f** “Average segmentation and **h-j** ”Segmentation error”). Prediction-only regions are shown in red, reference-only regions in green and overlap in yellow (**a,d,h**). Uncertainty panels show predictive entropy (**b,e,i**) and Monte Carlo dropout variance (**c,f,j**).

**Figure 6:**
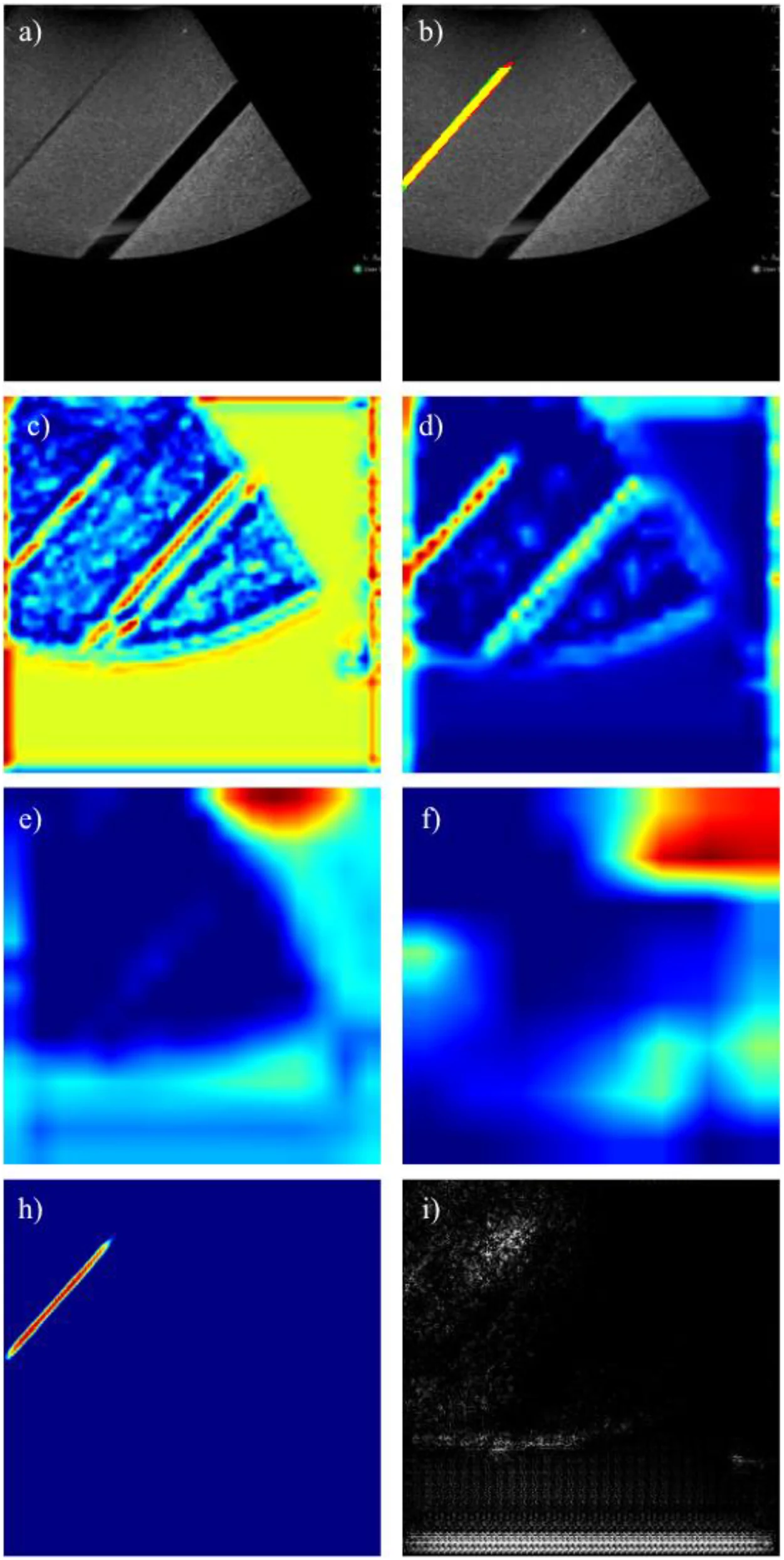
Good segmentation example: **(a)** raw image, **(b)** mask overlay, **(c–f)** Grad-CAM maps from the final residual block of encoder stages 1–4, **(h)** final decoder block, and **(i)** input-gradient saliency map.

**Figure 7:**
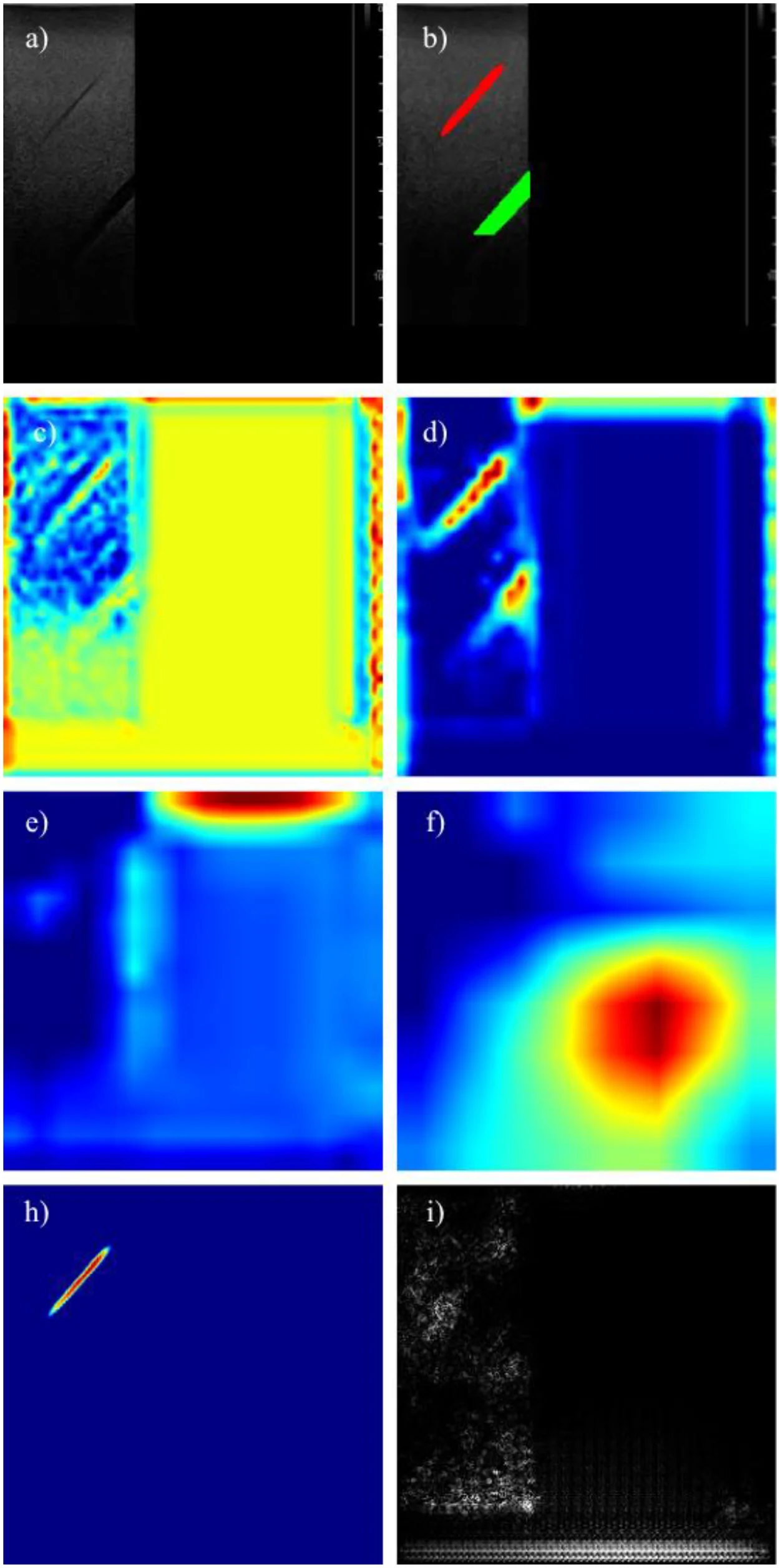
Erroneous segmentation example: **(a)** raw image, **(b)** mask overlay, **(c–f)** Grad-CAM maps from the final residual block of encoder stages 1–4, **(h)** final decoder block, and **(i)** input-gradient saliency map.

The examples were selected after inspection of the complete test-set outputs because each contained two visible pipes when only one pipe was the intended target. They were therefore illustrative examples rather than a prespecified representative sample. In the two successful examples, the model segmented the intended pipe and excluded the non-target pipe. Elevated predictive entropy and Monte Carlo dropout variance were nevertheless present around parts of the non-target structure.

In the good example, saliency was concentrated near pipe extremities, including the upper extremity used for the superficial visibility measurement (Figure 6i). In the erroneous example, the model segmented the non-target pipe, while elevated uncertainty and activation also overlapped parts of the intended target region (Figures 5h–j; Figures 7c,d). Earlier encoder maps retained finer spatial detail, whereas deeper encoder maps were coarser and the final decoder map reflected the reconstructed segmentation features. These visualisations indicate spatial sensitivity but do not establish that the highlighted regions causally determined the output.

### 3.4 Calibration and reliability analysis

Predictive entropy, Monte Carlo dropout variance and calibration metrics were broadly similar across the five folds (Table 2). Mean Monte Carlo dropout variance was below 0.01 in every fold, indicating limited variation across stochastic passes under the selected dropout configuration; this should not be interpreted as proof that the model had negligible epistemic uncertainty.

**Table 2:** Held-out uncertainty and calibration metrics by outer fold.

**Fold**	**Aleatoric**	**Epistemic**	**Brier**	**ECE**
1	0.19 [0.02]	<0.01	0.16 [0.06]	0.16 [0.06]
2	0.16 [0.02]	<0.01	0.15 [0.05]	0.17 [0.06]
3	0.12 [0.01]	<0.01	0.20 [0.05]	0.21 [0.05]
4	0.18 [0.02]	<0.01	0.18 [0.06]	0.18 [0.06]
5	0.14 [0.01]	<0.01	0.15 [0.04]	0.15 [0.04]
*Overall*	*0.16 [0.01]*	*<0*.*01*	*0.17 [0.02]*	*0.18 [0.02]*

Values are means with 95% confidence-interval half-widths in square brackets, calculated according to normal-theory. Predictive entropy is used as an approximation of data-related (aleatoric) ambiguity; MC variance is the pixel-wise variance across 20 Monte Carlo dropout passes and is used as an approximation of model-related (epistemic) uncertainty. ECE, expected calibration error.

A Spearman correlation matrix was used to describe associations among segmentation, uncertainty and calibration metrics (Figure 8). For descriptive interpretation, |*ρ*| ≥ 0.70 was labelled a strong association. Correlations were not interpreted as causal relationships or as direct evidence of prospective failure-prediction performance.

**Figure 8:**
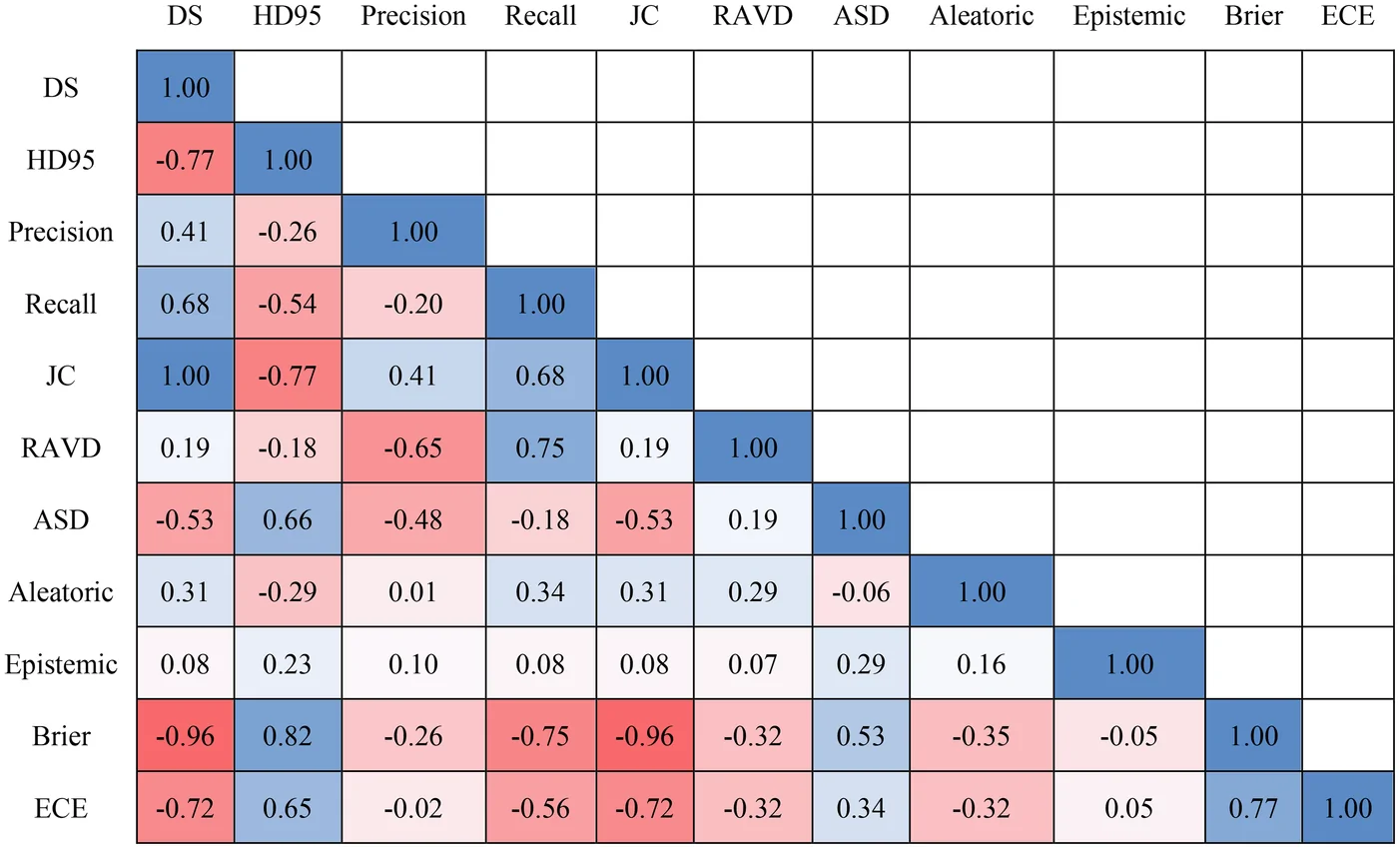
Spearman correlation matrix for segmentation performance, uncertainty and calibration metrics across held-out images.

DS was strongly negatively associated with the Brier score (*ρ* = −0.96) and ECE (*ρ* = −0.72), indicating that images with better overlap also tended to show closer agreement between predicted probabilities and reference labels. HD95 was positively associated with the Brier score (*ρ* = 0.82), so larger boundary errors tended to occur in images with larger probability errors.

DS and HD95 were also strongly associated (*ρ* = −0.77), as greater overlap generally corresponded to smaller boundary errors. The perfect correlation between DS and JC was expected because the two overlap metrics are monotonic transformations of one another.

Predictive entropy and Monte Carlo dropout variance showed only weak associations with DS and the other principal segmentation metrics (|*ρ*| < 0.4). Under this experimental configuration, neither uncertainty approximation therefore functioned as a strong image-level correlate of segmentation quality.

The mean Brier score was 0.17 ± 0.02 and the mean ECE was 0.18 ± 0.02. These values describe calibration within this dataset and should not be interpreted against a universal acceptability threshold. Their principal relevance here was the observed image-level association with reference-based segmentation performance.

Across folds, attention localisation showed the strongest mean positive association with DS in the final decoder block (mean *ρ* = 0.60), followed by encoder stage 2 (mean *ρ* = 0.51). Decoder attention entropy also showed a positive mean association with DS (mean *ρ* = 0.66), whereas the corresponding associations for the input-gradient saliency map were weak. The fold-to-fold variability was substantial, and no single layer provided a uniformly strong association in every fold (Figure 9).

**Figure 9:**
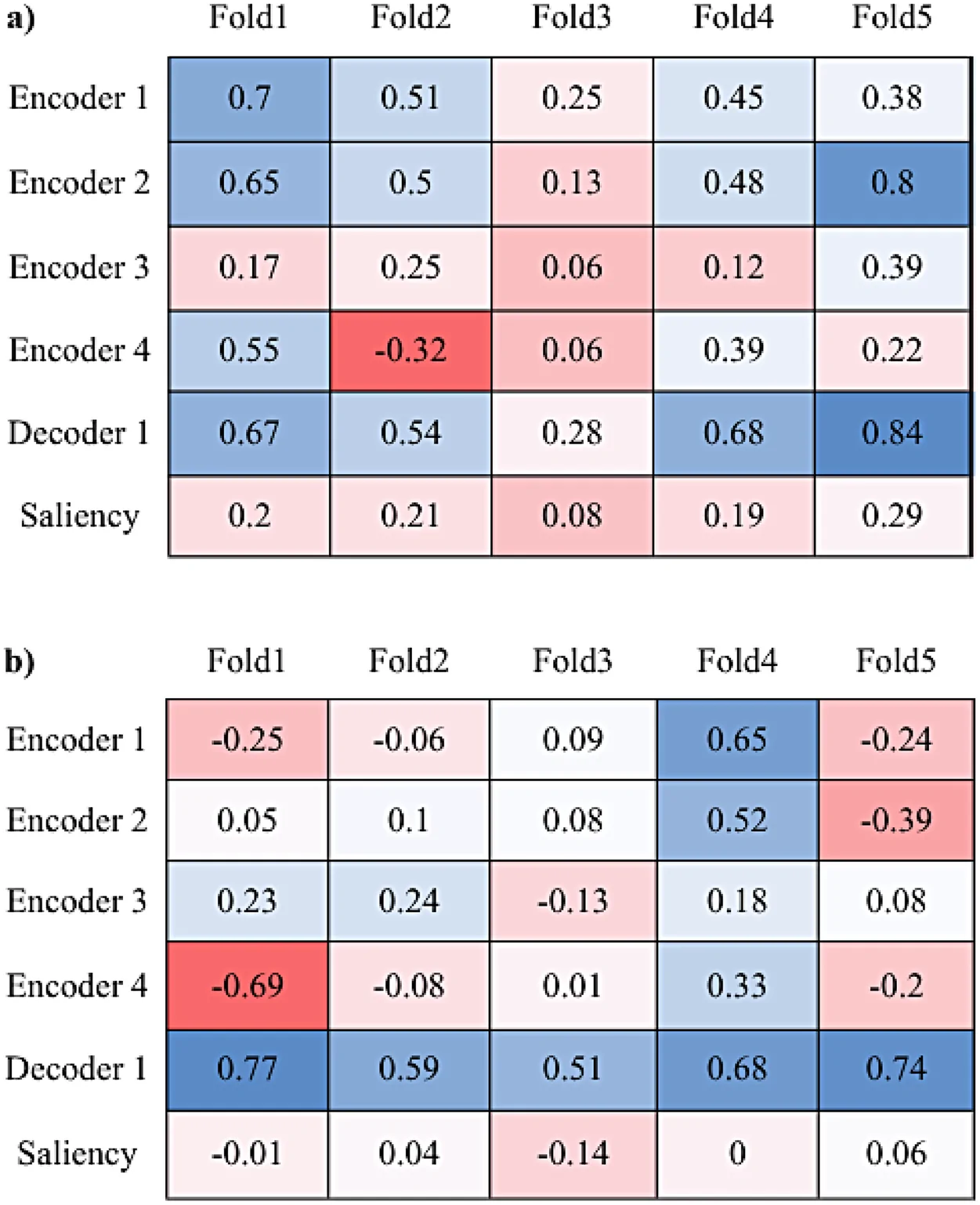
Fold-specific Spearman correlations between dice score and **(a)** attention localisation and **(b)** attention entropy for each grad-CAM layer and the input-gradient saliency map.

### 3.5 Failure mode analysis

Predictive-entropy, Monte Carlo dropout-variance and input-gradient saliency maps for two complete failure cases, in which no pipe was predicted despite a non-empty reference mask, are shown in Figure 10. Layer-wise Grad-CAM maps for the third complete failure are shown in Figure 11.

**Figure 10:**
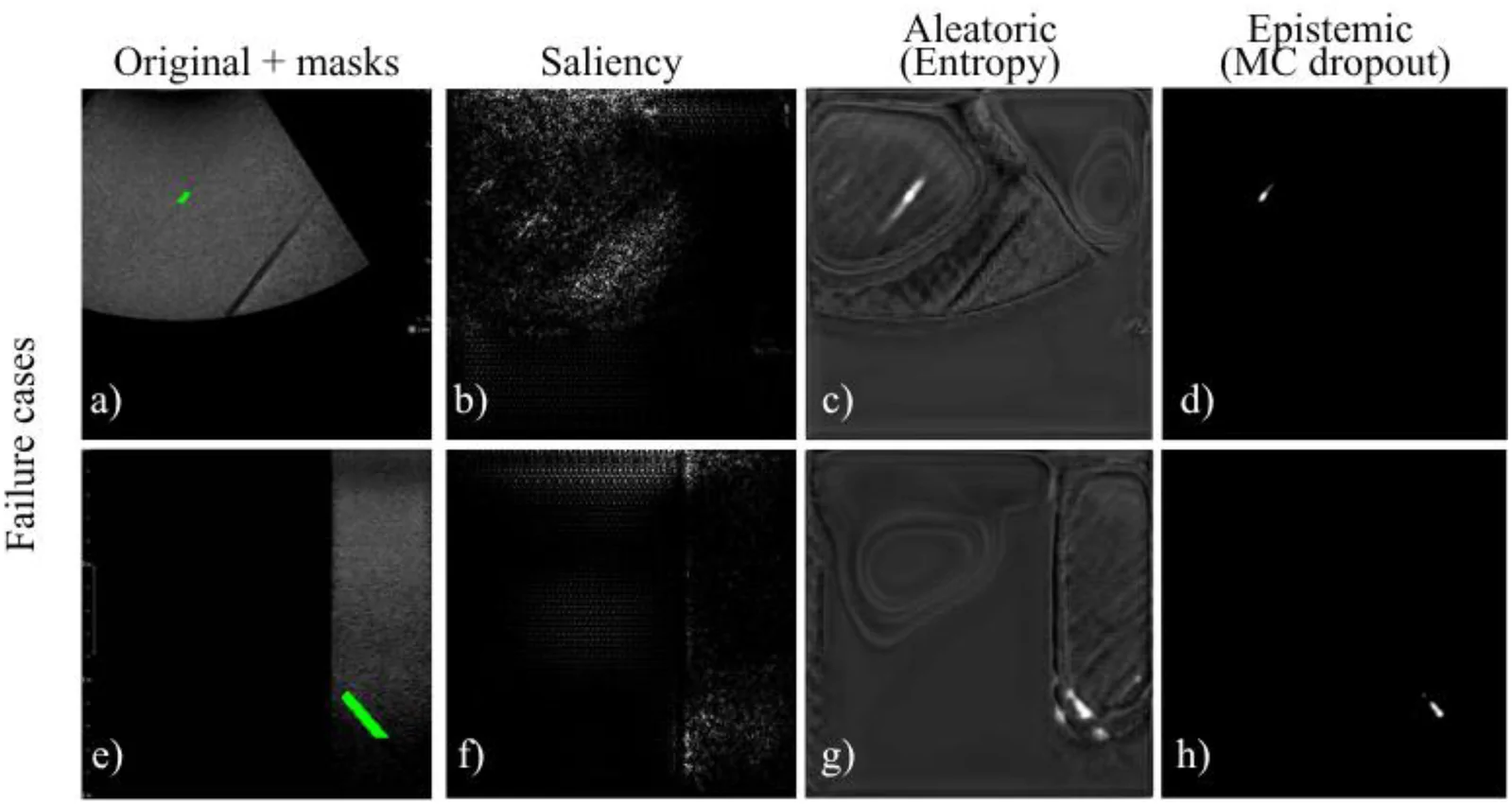
Predictive entropy (**c,g**), Monte Carlo dropout variance (**d,h**) and input-gradient saliency maps (**b,f**) for two complete failures. The reference pipe regions are shown in panels (**a**) and (**e**).

**Figure 11:**
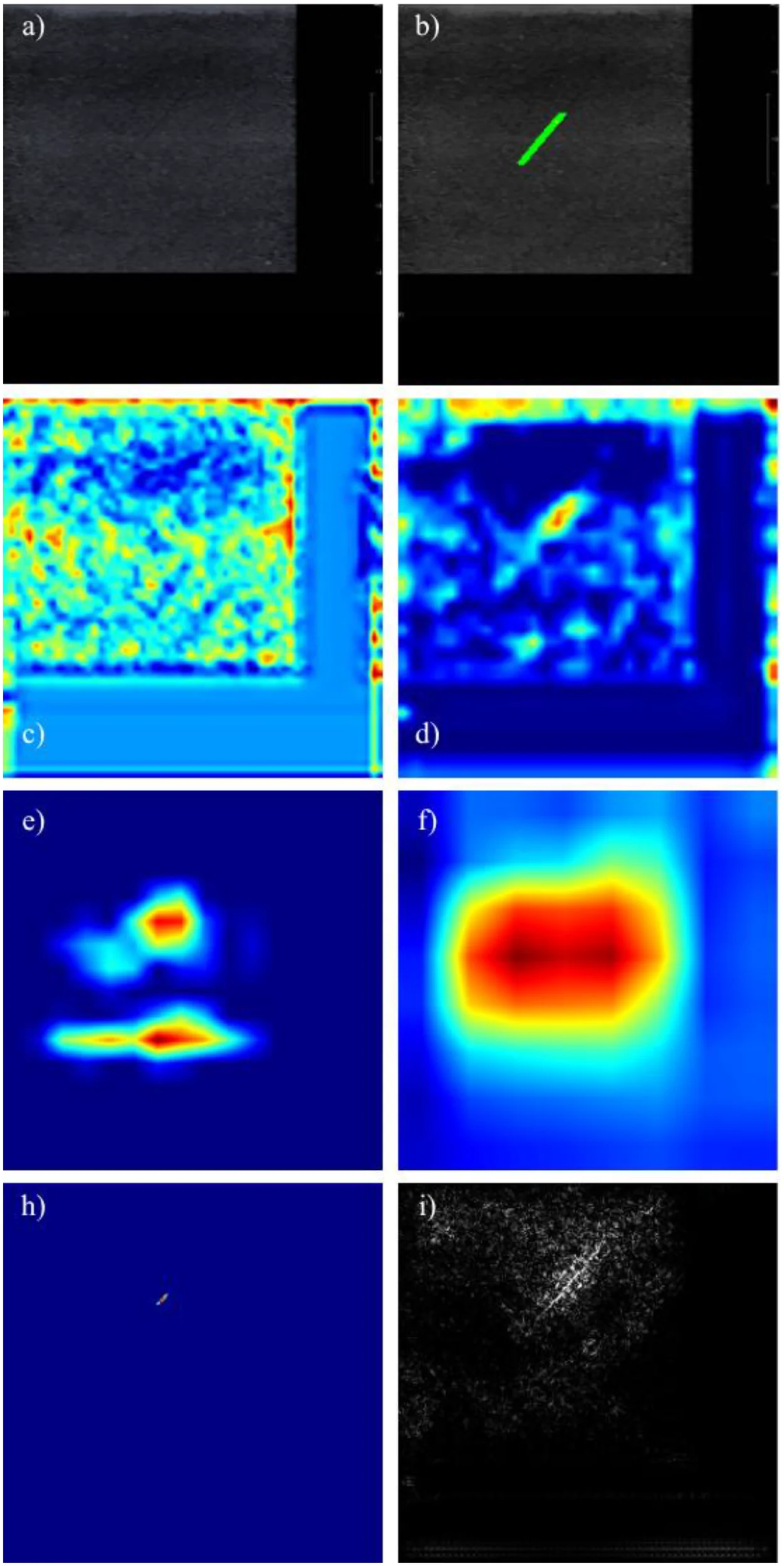
Catastrophic segmentation failure. **(a)** Raw image and **(b)** reference mask overlaid in green, **(c–f)** corresponding Grad-CAM maps from encoder blocks 1–4, **(h)** the last decoder block, and **(i)** the gradient-based saliency map.

For the two cases in Figure 10, elevated predictive entropy and Monte Carlo dropout variance overlapped parts of the reference pipe regions, whereas saliency activation in these regions was limited. In the third failure (Figure 11), activation overlapping the reference pipe was visible in encoder stages 2 and 3 and in the final decoder block, and the saliency map showed limited activation within the same region. These observations are consistent with sensitivity to pipe-related features that was insufficient to produce a foreground prediction, but they do not establish the internal causal mechanism of failure.

An exploratory image-level quality-control analysis was performed to assess whether outputs unsuitable for subsequent automated resolution integral measurement could be identified from the model output (see Table 3). Critical segmentation errors were separated into two categories. Complete segmentation failure was defined as the absence of predicted foreground when a reference pipe was present. These cases could be identified directly at inference from an empty predicted mask and were therefore handled using a deterministic quality-control flag (*n* = 3 in this dataset). Wrong-pipe errors were defined as non-empty predictions with no overlap with the corresponding reference pipe (Dice = 0; *n* = 4 in this dataset). Reference-free quality-control indicators were evaluated only on non-empty predictions (*n* = 159), where poor segmentation quality was retrospectively defined as Dice < 0.50 and/or HD95 > 20 pixels. A secondary analysis assessed the ability of these indicators to identify wrong-pipe segmentation errors specifically.

**Table 3:** Leave-one-fold-out performance of reference-free quality-control indicators for identifying poor-quality, non-empty segmentations.

**Method**	**Threshold**	**Sensitivity**	**Specificity**	**Review Rate**
Component Count Deviation ↑	0.00 ± 0.00	1.00 [0.86–1]	0.00 [0.00–0.03]	1.00
Predicted Area Deviation ↑	0.01 ± 0.01	1.00 [0.86–1]	0.00 [0.00–0.03]	1.00
Boundary Box Fill Fraction ↓	0.61 ± 0.06	0.96 [0.79–0.99]	0.00 [0.00–0.03]	0.99
Mean Entropy ↑	0.08 ± 0.00	0.96 [0.79–0.99]	0.01 [0.00–0.04]	0.99

Threshold direction is denoted by either ↑ for upper-limits or ↓ for lower limits. Low segmentation quality was defined retrospectively as DS < 0.50 and/or HD95 > 20 pixels. Only non-empty predicted masks were included (*n* = 159). Thresholds were selected using four folds and evaluated on the remaining held-out fold. Sensitivity and specificity represent pooled performance across all held-out predictions with 95% Wilson confidence intervals; threshold values are reported as mean ± SD across the five iterations.

Methods based on component count and predicted area deviations both achieved a sensitivity of 1.00 by identifying the 23 poor-quality cases, whilst methods based on the boundary box fill fraction and mean entropy only identified 22. However, these indicators showed insufficient specificity to support their use as prospective quality-control criteria in the present dataset. Their results were therefore interpreted as exploratory and were not proposed as deployable failure-detection methods (see Table 3). Of the four wrong-pipe errors with non-empty predictions, only the method based on component count deviation identified all cases whilst the remaining only identified three. Specificity was poor (<0.1) in all methods except for the one based on predicted area deviation, which achieved 0.58 [0.50–66].

Threshold-sensitivity curves and complete threshold sensitivity analysis results are provided in Supplementary Material B.

## 4 Discussion

This study combined segmentation metrics, calibration, uncertainty approximations, saliency, layer-wise Grad-CAM and exploratory quality-control indicators to evaluate a U-Net with a ResNet-18 encoder for EPP pipe segmentation. Held-out performance was consistent across the five image-level folds (overall DS = 0.81 ± 0.03), with three complete failures among 162 images. A single forward pass required 0.003 s on the GPU workstation and 0.036 s on the CPU-only laptop, compared with approximately 30 s for a manual image measurement (27). This represents an approximately 99.88% reduction in the segmentation component of the workflow, although the benchmark excluded the 20 stochastic passes and explanation-generation steps used in the full interpretability analysis.

The training and validation curves showed convergence in all folds, with greater validation variability and mild late divergence in some folds. Checkpoint selection limited the use of later, poorer-performing epochs, but these curves cannot demonstrate absence of overfitting. Moreover, the cross-validation design balanced images from each probe across folds and therefore evaluated new images acquired within the known probe and scanner distribution; it did not test generalisation to an entirely unseen probe, scanner or phantom. The reported cross-fold consistency should be interpreted within that narrower scope.

Calibration metrics were more closely associated with segmentation quality than the evaluated uncertainty approximations. DS was strongly associated with both the Brier score and ECE, and HD95 was strongly associated with the Brier score. These relationships indicate that poorer segmentations tended to contain larger probability errors or poorer probability-frequency agreement. However, calibration and segmentation metrics were calculated against the same reference masks, so the correlations are not independent evidence that calibration can prospectively identify failure. Correlation also does not establish predictive usefulness or causality. Brier score and ECE are therefore most appropriately interpreted here as complementary retrospective measures of probabilistic behaviour.

Predictive entropy and Monte Carlo dropout variance showed weak associations with segmentation performance. The low variance across stochastic passes may reflect the controlled phantom testing dataset, which was broadly similar to the training data. However, it may also depend on the dropout probability, the placement of dropout before the decoder and the use of 20 forward passes. Monte Carlo dropout was selected because it could be incorporated into a single trained network with modest implementation and storage requirements, but it is only an approximate uncertainty method. Deep ensembles, test-time augmentation, Bayesian neural networks or evidential approaches may produce different uncertainty estimates and should be compared in future work before broader conclusions are drawn.

The visual analyses provided complementary, but necessarily limited, information. In selected multi-pipe images, elevated entropy, variance or activation overlapped both target and non-target structures, including in complete failures. The final decoder Grad-CAM maps showed the strongest mean associations with Dice score for attention localisation and entropy, although these associations varied across folds. Earlier encoder maps retained finer spatial detail, whereas later maps were coarser because of progressive downsampling. Input-gradient saliency showed comparatively weak associations with Dice score. Importantly, these maps represent sensitivity or activation, not causal explanations of the decision process. The examples were selected retrospectively, and no model-parameter or label-randomisation sanity tests were performed; the qualitative findings should therefore be considered hypothesis-generating.

The present results support a simple deterministic check for empty model outputs and flagging of low-quality segmentations requiring review by the user. However, the study did not identify a sufficiently sensitive reference-free indicator for detecting more subtle, non-empty segmentation errors. Prospective quality control of low-quality but non-empty predictions therefore remains an area for future investigation.

For the intended medical-physics workflow, a future quality-control interface could display the predicted pipe mask and flag outputs with an empty or implausible foreground region for manual review before the resolution integral is calculated. Probability calibration could be monitored retrospectively on labelled quality-assurance datasets, but it would not be available as an image-specific reference-free check in routine use. Uncertainty maps may help developers investigate recurring failure patterns, although presenting them directly to users would require evidence that they improve decisions rather than simply increasing cognitive burden. The framework should therefore be viewed as a development and audit tool for EPP automation, not as a validated clinical decision-support system.

Several limitations remain. The dataset was small and contained controlled phantom images rather than clinical ultrasound scans. Consequently, it lacked anatomical variability, pathology, patient motion, and many acquisition artefacts. The controlled phantom setting nevertheless supports the intended use for scanner calibration and standardised assessment, but it does not permit conclusions regarding performance on clinical ultrasound data. Only one architecture was evaluated in the present experiments; the earlier architecture comparison summarised in Supplementary Material A used a different split and does not establish that the interpretability findings are architecture-independent. External validation on a separate phantom or scanner was not performed. The reference masks were produced by one operator from measurements acquired by another, and agreement in derived R values was high, but independent boundary-level inter-rater reliability was not assessed. Although no repeated frames or duplicate acquisitions exist for a given scanner-probe-pipe-position combination, image pairs from both position of the same physical pipe imaged with the same scanner and probe could be found in both training and testing sets. As these images could potentially have shared characteristics, a grouped cross-validation sensitivity analysis by scanner-probe-pipe should be performed to investigate the impact of the potential dependence. Finally, training time, peak memory use and the full computational overhead of Monte Carlo dropout and explanation generation were not benchmarked.

## 5 Conclusion

Within this controlled EPP dataset, the evaluated U-Net with a ResNet-18 encoder achieved consistent held-out segmentation performance and a low complete-failure rate. Calibration metrics were more strongly associated with reference-based segmentation quality than predictive entropy or Monte Carlo dropout variance, while saliency and layer-wise Grad-CAM provided descriptive information about selected errors that was not captured by overlap metrics alone. However, the reference-free quality-control indicators lacked specificity, and the visualisations do not constitute causal explanations. The framework is therefore useful as a proof of concept for auditing EPP segmentation models, but independent validation, alternative architectures and uncertainty methods, and prospective threshold evaluation are required before broader or deployable reliability claims can be made.

## Data Availability

The datasets presented in this study can be found in online repositories. The names of the repository/repositories and accession number(s) can be found in the article/Supplementary Material.
