# Supplementary material for: Beyond segmentation accuracy: understanding model reliability and failure modes in ultrasound image analysis
Source: Front Med Technol. 2026 Sep 23;8:1918432. doi: 10.3389/fmedt.2026.1918432 (PMC13642736; doi:10.3389/fmedt.2026.1918432)
Supplement: Supplementary file 1 [file Supplementaryfile1.pdf]

6 Supplementary materials

Supplementary material A. *a) Training and b) testing segmentation performance metrics for model architectures previously evaluated for Edinburgh Pipe Phantom measurements.*

The dataset[1] from MacGillivray et al. 2010[2] was split according to a 70-20-10 split in training, validation and testing subsets to compare candidate architectures for automated measurements on the Edinburgh Pipe Phantom. Dice score and mean Hausdorff distance were calculated during training and testing. Application-specific performance was also assessed using the percentage difference between machine-learning-derived pipe depths and the reference values obtained by agreement among four observers. These earlier results are provided to explain the selection of the ResNet-18 U-Net used in the present study; they are not a new ablation analysis under the current five-fold design.

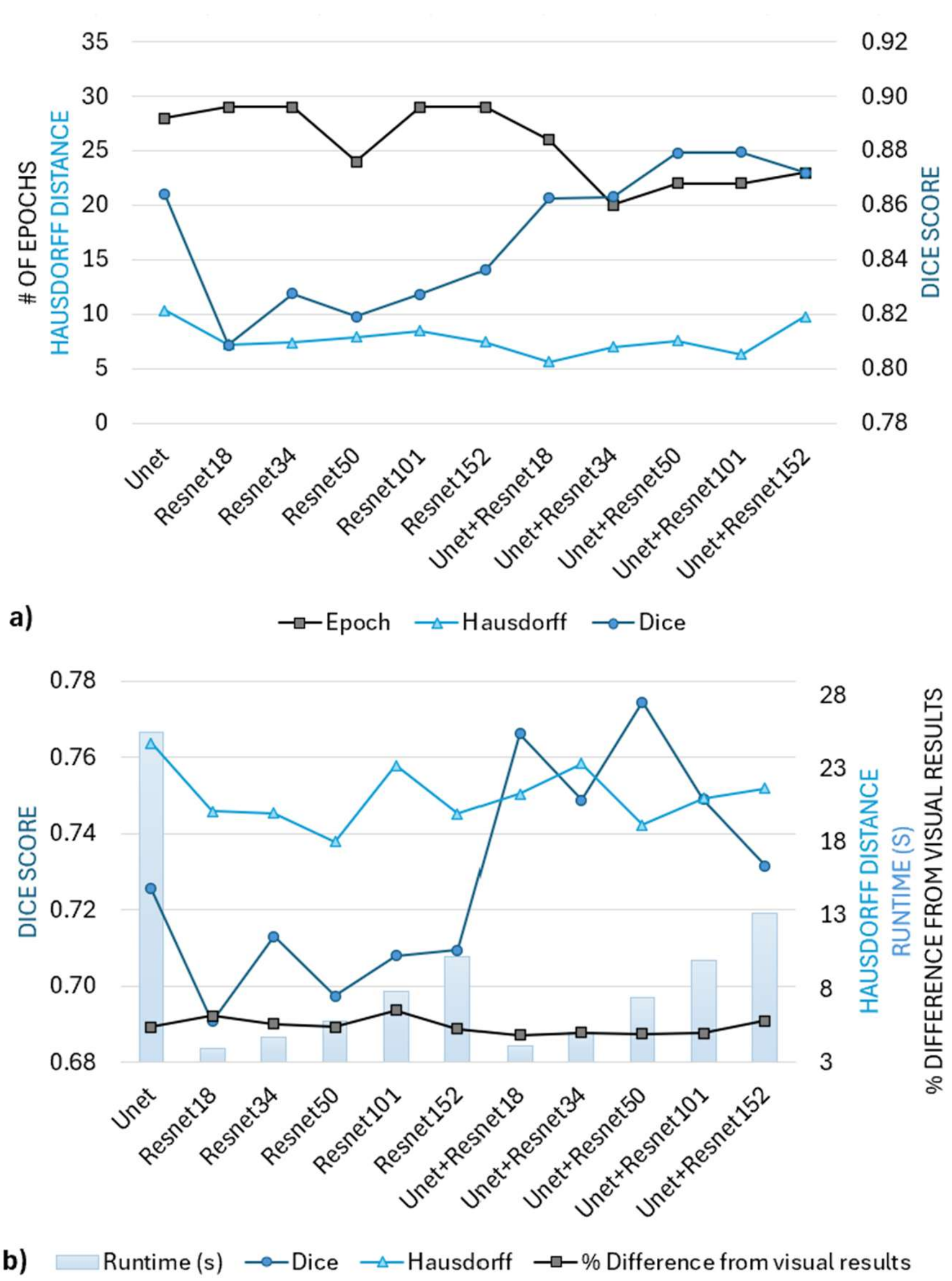

[1] Bricout A. Segmentation of Edinburgh Pipe Phantom scans for resolution integral measurements. Mendeley Data 2025;V1. https://doi.org/10.17632/ZJWZ8D5T5V.1.

[2] MacGillivray TJ, Ellis W, Pye SD. The resolution integral: Visual and computational approaches to characterizing ultrasound images. Phys Med Biol 2010;55:5067–88. https://doi.org/10.1088/0031-9155/55/17/012.

Supplementary material B. *Threshold sensitivity results and plots for the exploratory image-level quality-control analysis using a) Component count deviation, b) Predicted area log deviation, c) Boundary-box fill fraction, and d) mean predictive entropy (estimated aleatoric uncertainty).*

Table B. Complete sensitivity threshold analysis for all considered prospective metrics, to detect poor quality and erroneous segmentations.

Target	Metric	TP	FP	TN	FN	Sensitivity	Specificity	Precision	Balanced Accuracy	Review Rate	Threshold
Poor Quality	Component Count Deviation	23	136	0	0	1.00 [0.86-1]	0.00 [0.00-0.03]	0.14	0.50	1.00	0.00 ± 0.00
Poor Quality	Largest Component Fraction	23	136	0	0	1.00 [0.86-1]	0.00 [0.00-0.03]	0.14	0.50	1.00	1.00 ± 0.00
Poor Quality	TopBottom LogRatio Deviation	22	136	0	1	0.96 [0.79-0.99]	0.00 [0.00-0.03]	0.14	0.48	0.99	0.16 ± 0.11
Poor Quality	PredArea LogDeviation	23	136	0	0	1.00 [0.86-1]	0.00 [0.00-0.03]	0.14	0.50	1.00	0.01 ± 0.01
Poor Quality	Border Foreground Fraction	23	136	0	0	1.00 [0.86-1]	0.00 [0.00-0.03]	0.14	0.50	1.00	0.00 ± 0.00
Poor Quality	BBoxFill Fraction	22	136	0	1	0.96 [0.79-0.99]	0.00 [0.00-0.03]	0.14	0.48	0.99	0.61 ± 0.06
Poor Quality	Touches Border	23	136	0	0	1.00 [0.86-1]	0.00 [0.00-0.03]	0.14	0.50	1.00	0.00 ± 0.00
Poor Quality	Mean Entropy	22	135	1	1	0.96 [0.79-0.99]	0.01 [0.00-0.04]	0.14	0.48	0.99	0.08 ± 0.00
Wrong Pipe	Component Count Deviation	4	144	11	0	1.00 [0.51-1.00]	0.07 [0.04-0.12]	0.03	0.54	0.93	0.00 ± 0.00
Wrong Pipe	Largest Component Fraction	4	155	0	0	1.00 [0.51-1.00]	0.00 [0.00-0.02]	0.03	0.50	1.00	1.00 ± 0.00
Wrong Pipe	TopBottom LogRatio Deviation	4	145	10	0	1.00 [0.51-1.00]	0.06 [0.04-0.11]	0.03	0.53	0.94	4.02 ± 0.20
Wrong Pipe	PredArea LogDeviation	3	65	90	1	0.75 [0.30-0.95]	0.58 [0.50-0.66]	0.04	0.67	0.43	0.60 ± 0.32
Wrong Pipe	Border Foreground Fraction	4	155	0	0	1.00 [0.51-1.00]	0.00 [0.00-0.02]	0.03	0.50	1.00	0.00 ± 0.00
Wrong Pipe	BBoxFill Fraction	3	146	9	1	0.75 [0.30-0.95]	0.06 [0.03-0.11]	0.02	0.40	0.94	0.48 ± 0.17
Wrong Pipe	Touches Border	4	155	0	0	1.00 [0.51-1.00]	0.00 [0.00-0.02]	0.03	0.50	1.00	0.00 ± 0.00
Wrong Pipe	Mean Entropy	3	149	6	1	0.75 [0.30-0.95]	0.04 [0.02-0.08]	0.02	0.39	0.96	0.08 ± 0.01

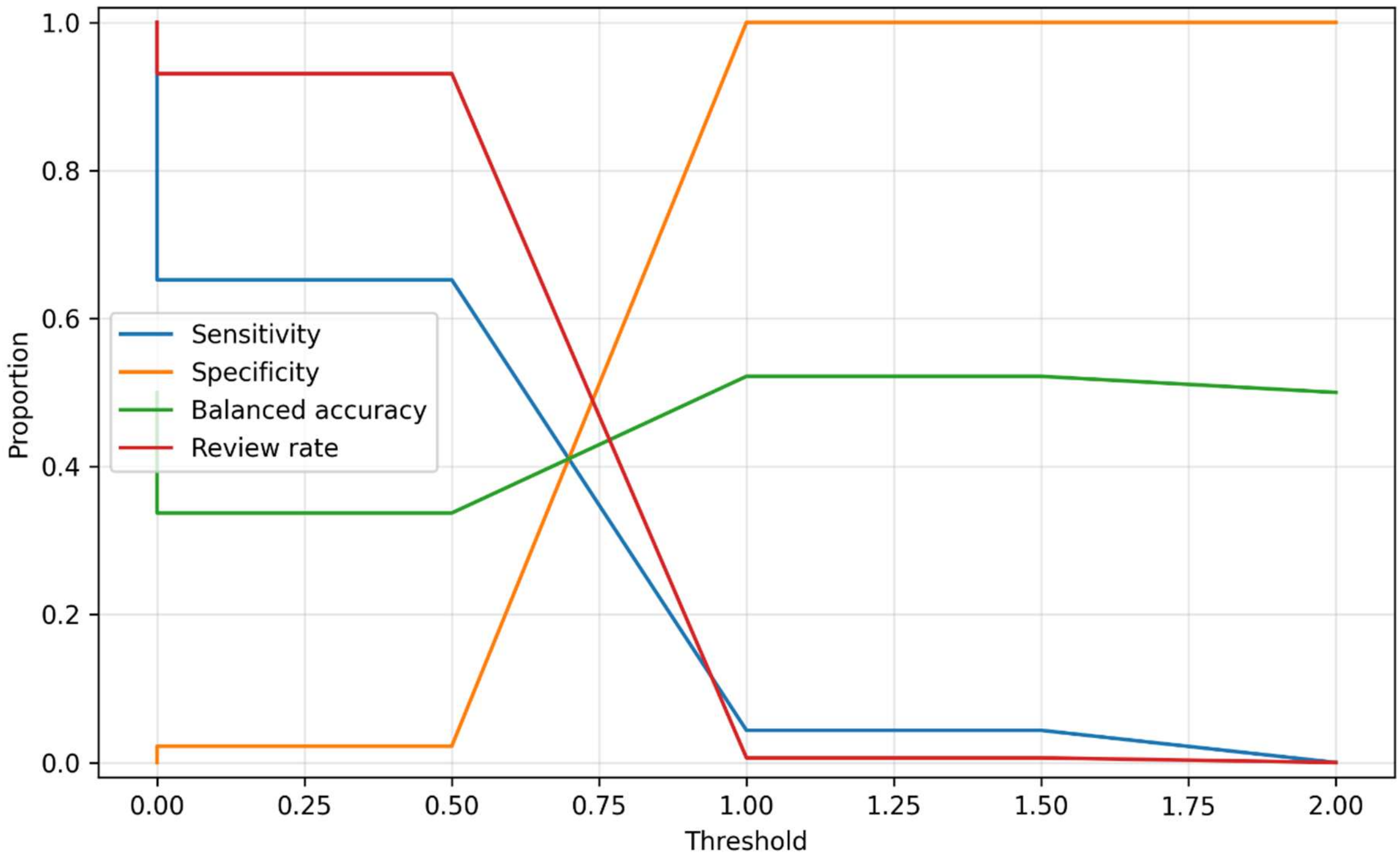
1.0
0.8
0.6
0.4
0.2
0.0
Proportion
Sensitivity
Specificity
Balanced accuracy
Review rate
0.00
0.25
0.50
0.75
1.00
1.25
1.50
1.75
2.00
Threshold

a)

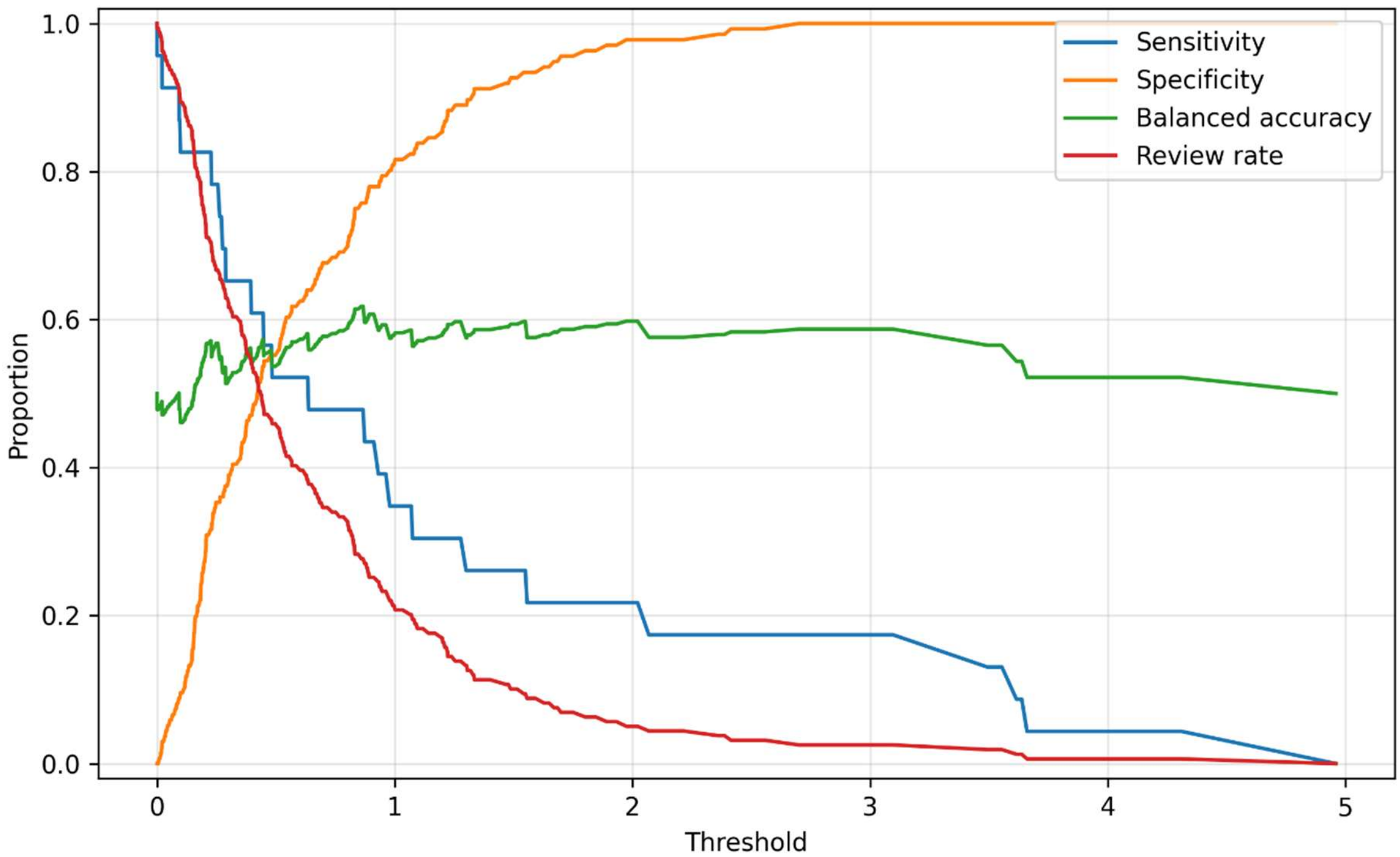
1.0
0.8
0.6
0.4
0.2
0.0
Proportion
Sensitivity
Specificity
Balanced accuracy
Review rate
0
1
2
3
4
5
Threshold

b)

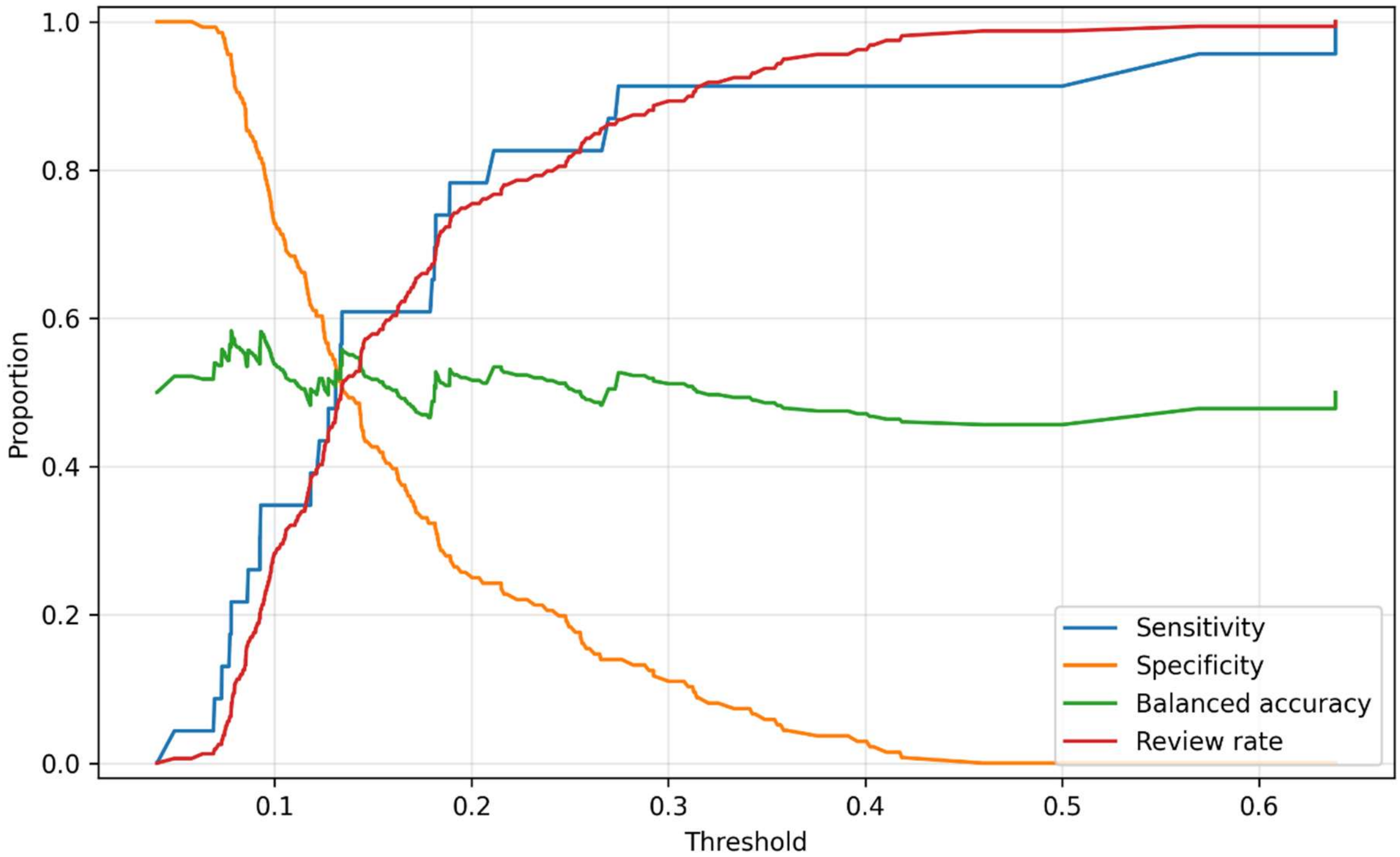
Proportion
Threshold
Sensitivity
Specificity
Balanced accuracy
Review rate

c)

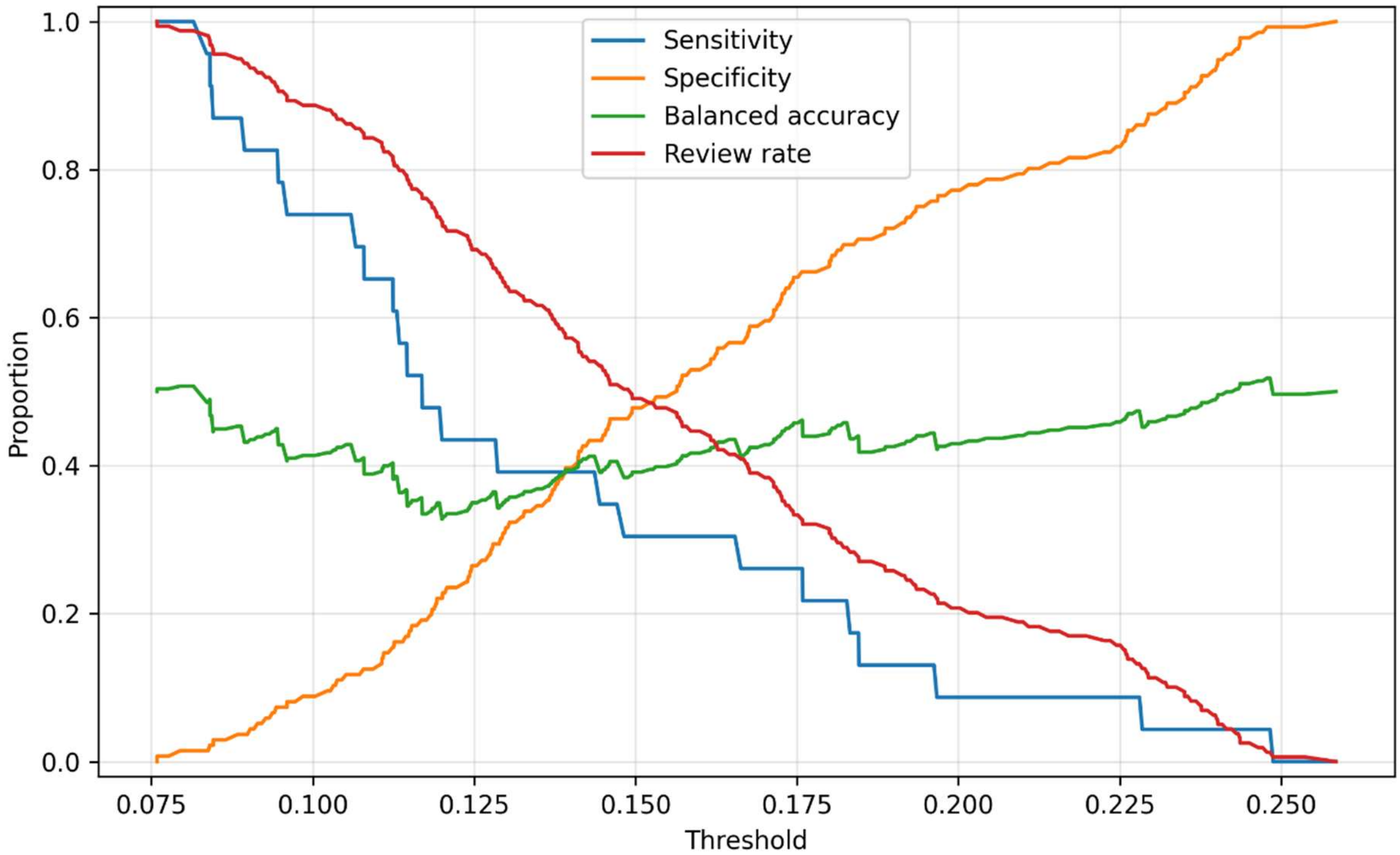
Proportion
Threshold
Sensitivity
Specificity
Balanced accuracy
Review rate

d)
